# The effect of Montreal’s supervised consumption sites on injection-related infections among people who inject drugs: An interrupted time series

**DOI:** 10.1371/journal.pone.0308482

**Published:** 2024-08-27

**Authors:** Jihoon Lim, Dimitra Panagiotoglou

**Affiliations:** Department of Epidemiology, Biostatistics, and Occupational Health, McGill University, Montreal, Quebec, Canada; Keck School of Medicine, University of Southern California, UNITED STATES OF AMERICA

## Abstract

**Background:**

Between June and November 2017, four supervised consumption sites (SCS) began operating in Montreal, Quebec. Earlier studies on SCS focused on examining their effects on blood-borne viral infections and overdose mortality. Our objective was to examine the effect of Montreal’s SCS on the incidence, health service use and outcomes of injection-related infections (IRI) in people who inject drugs.

**Methods:**

We used Quebec’s provincial administrative health data to identify people who inject drugs in Montreal and calculated the incidence of IRI in this population between December 2014 and December 2019. We conducted a retrospective, population-based interrupted time series to estimate the effect of Montreal’s four SCS on the monthly incidence rates of IRI-related hospitalizations, emergency department (ED) visits, physician visits, and mortality. We also examined the effects of SCS on average length of IRI-related hospitalizations and incidence of hospitalizations involving surgery.

**Results:**

The average age of Montreal’s people who inject drugs was 41.84 years, and 66.41% were male. After the implementation of SCS, there was a positive level change in the incidence of hospitalizations (0.97; 95% confidence interval [CI]: 0.26, 1.68) for IRI. There was also a significant post-intervention decline in hospitalization trends (-0.05; 95% CI: -0.08, -0.02), with modest trend changes in ED visits (-0.02; 95% CI: -0.05, 0.02). However, post-intervention changes in level (0.72; 95% CI: -3.85, 5.29) and trend (0.06; 95% CI: -0.23, 0.34) for physician visits remained limited. SCS had no effect on the average length of hospitalizations, but there was a decreasing post-intervention trend in hospitalizations involving surgery (-0.03; 95% CI: -0.06, 0.00).

**Conclusion:**

Following the opening of the SCS, there was a moderate decline in the rate of hospitalizations to treat IRI, but the impact of the sites on the rate of physician visits remained limited. These findings suggest that SCS may mitigate the incidence of more serious and complicated IRI over time.

## Introduction

Injection drug use remains a serious public health concern. Associated behaviours including injection frequency and equipment sharing elevate the risk of HIV and hepatitis C virus (HCV) infections [1–3], while a toxic supply increases the risk of drug poisoning [4]. In Quebec, Canada although people who inject drugs account for an estimated 0.27% of the total population [5], they represent 13% and 63% of people living with HIV and HCV, respectively [6]. Meanwhile, the number of fatal overdoses in the province has increased over the past decade, with a record number of deaths reported in 2022 (541 cases; 6.3 cases per 100,000 population) [7]. However, harms associated with injection drug use extend beyond blood borne infections and poisonings.

Injection-related infections (IRI), such as skin, soft tissue, and vascular infections, are pathological conditions caused by bacterial invasion of skin and subcutaneous soft tissues at the drug injection site. Although some cases are superficial, patients with IRI often experience pain, swelling, and the formation of lesions and bullae [8]. Early treatment strategies by people who inject drugs include mechanical drainage, warm compress, and antibiotics obtained from unregulated markets [9, 10]. While some cases resolve on their own or respond to self-treatment efforts, without formal medical care, IRI can progress into more severe complications including necrotizing fasciitis, sepsis, and endocarditis [8, 11–13], and may lead to debilitating amputations or deaths [14–17].

For people who inject drugs, IRI contribute to significant morbidity and remain among the leading causes of emergency department visits and hospitalizations, globally [18–25]. Aside from harms to individuals, IRI place a substantial economic burden on healthcare systems. For example, in 2021, the average cost and length of stay per adult (18–59 years) hospitalized in Quebec for cellulitis was $8,371 and 4.8 days, and $8,090 and 3.9 days for abscess, respectively [26]. Efforts to diagnose and treat IRI early may reduce the risk of severe complications, improving patients’ quality of life and curtailing costs to the healthcare system.

As part of its harm reduction strategy, the city of Montreal, Quebec implemented four supervised consumption sites (SCS) in 2017. Best known as legally sanctioned sites where people can inject pre-obtained drugs under the supervision of medically trained staff, SCS may also offer drug checking, clean injecting equipment, medical and social services (e.g. HIV rapid testing, and housing and employment support), treatment referrals, and education on safe injection practices and wound care [27]. While studies have repeatedly demonstrated the (cost-) effectiveness of SCS on HIV and HCV [28–31], their effects on opioid-related emergency department visits, hospitalizations, and mortality remains mixed [32, 33]. Nevertheless, SCS serve as a physically safe environment away from street crime and harassment [34], where people who inject drugs may receive health care [35]. Weekly SCS use is associated with lower risk of all-cause mortality compared to more sporadic use [36], and one study observed hospital length of stays for cutaneous injection-related infections are substantially shorter for SCS clients compared to peers who do not visit sites [37]. Together, these findings suggest the benefits of SCS may extend beyond overdose reversal and prevention of infectious disease transmission.

Despite IRI’s high morbidity burden, and indication that SCS may affect IRI outcomes for people who inject drugs, no studies have looked at the intervention’s population-level effects on these infections. In this study, we aim to assess the impact of SCS on IRI health outcomes and related health service use for people who inject drugs in Montreal. We hypothesize that implementation of SCS improved IRI outcomes as people who inject drugs gained access to medically-informed advice for infections.

## Materials and methods

### Setting

Montreal is Quebec’s largest city, with a population of around 1.7 million and an estimated 4,000 people who are currently injecting drugs [38–40]. In June 2017, two fixed sites (Cactus, in downtown Montreal; Dopamine, in Hochelaga-Maisonneuve neighbourhood) along with one mobile site (L’Anonyme) became the first legally sanctioned SCS in the province [41, 42]. A fourth SCS (Spectre de Rue) started its operations in November 2017 [43, 44].

Approximately 97% of Quebec residents are insured by the province’s universal health insurance, Régie de l’Assurance Maladie du Québec (RAMQ). The remaining three percent include foreign tourists and international students not eligible for insurance, as well as federally insured refugee claimants, military personnel, and First Nations and Inuit peoples [45].

### Data sources

We used data from the © Government of Québec (Research file publication date: 2009–2019; Data use approval date: 20 September 2021) to develop the study cohort (original text in French under S1 Text). The data came from seven de-identified and linked provincial administrative health databases provided by the Québec Ministère de la Santé et des Services Sociaux, and RAMQ: (1) *Maintenance et exploitation des données pour l’étude de la clientèle hospitalière* (MED-ECHO), (2) *Services médicaux rémunérés à l’acte* (“Physician Claims”), (3) *Banque de données communes des urgences* (BDCU), (4) *Bureau du Coroner du Québec* (“Vital Statistics/Mortality”), (5) *Services pharmaceutiques* (“Public prescription drug plan” [PPDP]), (6) *Fichier sur les périodes d’admissibilité à l’assurance médicaments* (“Drug prescription programs and plans file”), and (7) *Registre des événements démographiques* (“Registered persons file”).

Datasets 1–4 included records of hospitalizations, physician visits, emergency department (ED) visits, and deaths, respectively. These data were used to identify people who inject drugs and ascertain outcomes relevant to the study. Datasets 5–6 contained information on prescription drug coverage and dispensation details, and were used to identify opioid agonist treatment prescription dispensations for people covered by the province’s public drug insurance program (representing approximately 44% of the population and comprised of low income, elderly, or otherwise lacking employer insurance coverage individuals) [46, 47]. Dataset 7 contained demographic variables. The datasets included records from 1 January 2010 to 31 December 2019, with a one-year wash-in period (1 January 2009–31 December 2009). Further information regarding these datasets can be found by visiting the *Institut de la Statistique du Québec* (ISQ) webpage at: https://statistique.quebec.ca/services-recherche/donnees/administratives [48]. All inferences, opinions, and conclusions drawn in this publication are those of the authors, and the © Government of Québec is not responsible for the compilations or the interpretation of the results produced using the research files (original text in French under S1 Text).

### Study population

We used a validated algorithm for Canadian administrative data to identify people who inject drugs and live in Montreal, Canada [49]. We defined people who inject drugs as adults (≥ 18 years of age) who had two medical visits (physician visits or emergency department visits), one hospitalization with diagnostic codes with disorders suggestive of injection drug use, or a record for opioid agonist treatment (OAT) (S1 Table). Cohort entry was determined using the first date of medical visit, hospitalization, or OAT dispensation. Montreal residency was determined using the individual’s socio-sanitary region code (‘06’ for the entire Montreal Island) in the “Drug prescription programs and plans” file. All cohort members were followed up until administrative censoring (December 31, 2019), end of registration with the RAMQ (i.e., moved out of province), or death, whichever occurred first.

### Study design

We used an interrupted time series (ITS) study design to examine the effects of Montreal’s SCS on IRI outcomes and health service use among people who inject drugs.

We assumed the rates of IRI in the pre-intervention period were a suitable counterfactual to the post-intervention period [50]. Where these assumptions hold true, the ITS design enables evaluation of the effect of SCS through comparison of the observed post-intervention outcome rates with the counterfactual (expected) post-intervention rates. In addition, although Montreal adopted other harm reduction interventions during the observation period (e.g., Good Samaritan Drug Overdose Act [May 2017] and paramedic use of naloxone [November 2017]), these were unlikely to have influenced IRI outcomes because they were specific to overdose event response [51, 52]. By modelling the underlying time trends, the ITS controls for the within-group time-fixed characteristics, secular trends, and random fluctuations from one time window to the next [53, 54].

The study period consisted of 30 “pre-intervention” monthly intervals (12/2014-05/2017) and 25 “post-intervention” intervals (12/2017-12/2019). We censored on the months between the two SCS openings (June 2017 and November 2017) because sites were launched mid-month [44, 55, 56], and segmented regression with a short “phase-in” period (here, July 2017-October 2017) would lead to unstable estimates of the impact of the intervention [57].

We chose December 2014 as the start of the observation period because designs with a balanced number of pre- and post-intervention time intervals have more power than those with an unbalanced periods [58, 59]. However, we utilized data from as early as January 2009 to identify people who inject drugs because cohort studies indicate many (74.2%) in the province have injected drugs for six years or longer [6]; enabling us to capture a larger and more stable segment of the population at risk of IRI.

### Outcome measures

The four primary outcomes for this study were the incidence rates (number of cases per 1,000 person-months) of IRI-related hospitalizations, ED visits, physician visits, and mortality, in our open cohort of people who inject drugs in Montreal. To ascertain IRI, we used the *Maintenance et exploitation des données pour l’étude de la clientèle hospitalière* (MED-ECHO), *Banque de données communes des urgences* (BDCU), *Services médicaux rémunérés à l’acte* (“Physician Claims”), and *Bureau du coroner du Québec* (“Vital Statistics/Mortality”) databases with the ICD-9/10 codes specified in S2 Table.

For each monthly interval (12/2014-12/2019), we calculated the number of IRI episodes and divided by the number of person-months for that period to obtain the incidence rate. We considered one or more encounters with the same diagnostic codes separated by less than 7 days in the MED-ECHO, BDCU, and Physician Claims as a single IRI episode based on a definition previously employed [22]. Individuals who experienced repeat IRI (i.e., infections recorded with 7 or more days between) had all events within the observation period included in the analysis. This approach ensured a more clinically relevant and interpretable measure of disease burden in the study population [60].

As secondary outcomes, we examined SCS’ impact on the average duration of hospital stay and rates of hospitalizations that involved surgical interventions. We defined surgical interventions based on the Canadian Classification of Health Interventions (CCI) procedure codes with the Section and Groups 1YA-1YZ, 2YA-2YZ, and 3YL-3YZ (Interventions on the Skin, Subcutaneous Tissue and Breast) with the qualifiers: ‘LA’, ‘DA’, ‘CA’, ‘JA’, and ‘HA’ [61, 62].

### Data preparation and analysis

We constructed a segmented regression model with a harmonic function to account for seasonal variations in IRI [63–65].


$$Y_{jt}=\beta_{0}+\beta_{1}Time_{t}+\beta_{2}Level_{j}+\beta_{3}Trend_{jt}+\beta h\left(\cdot \right)+\epsilon$$ (1)

For the intervention *j*, at time *t*, *Y*_*jt*_ is the outcome at time *t*. *Time*_*t*_ denotes time elapsed since the start of study and represents the frequency with which *Y*_*t*_ is measured. *Level*_*j*_ denotes the binary intervention variable, where 1 is after the last SCS was implemented (recall, November 2017) and 0 is before the first three opened (here, June 2017). *Trend*_*jt*_ represents the elapsed time since the intervention was fully implemented (November 2017). The *h*(⋅) term represents the harmonic function added to account for seasonal trends in IRI incidence.

*β*_0_ denotes the rate of outcome events at baseline (*t* = 0). *β*_1_ represents the pre-existing trend (slope) in the incidence rate for the outcome. *β*_2_ indicates the level change in outcome or the difference in the level of *Y*_*t*_ once SCS were implemented, relative to the counterfactual that expects no level change between the pre-intervention period and the post-intervention period. *β*_3_ represents the trend change in the outcome (i.e., slope change) from the expected trend (i.e., assuming no change in trend post- compared with pre-implementation). *β* represents the coefficients for the harmonic terms added to the regression model.

For the main impact model, we hypothesized that there would be immediate intervention level and trend effects due to the following reasons: (1) The opening of SCS was highly anticipated [41, 66]; (2) There were nearly 1,200 visits to these sites in the first month of their operations [56]; and (3) IRI are conditions with rapid onset that can manifest and progress within a short time frame [67].

For secondary analysis, we conducted the same analysis but with the outcome restricted to the incidence of skin and soft tissues infections (SSTI). We chose to focus on this subset of infections because they are the most commonly occurring forms of injection-related infections for people who inject drugs [68–70].

For sensitivity analyses, we re-ran the model with two different approaches. First, we repeated the analysis using June 2017 as the implementation date (i.e., only censored the month of June, instead of the months June to November as above). This approach assumes the opening of the first three sites cause the effect. This was because the fourth site (Spectre de Rue) accommodates only four people at a time with limited hours of operation (from 9:30 AM to 6 PM), for an estimated 30 injections per day [44]. Second, we repeated the same analysis, censoring only on the November 2017 time window. For this analysis, we assumed accumulation of effects from the fourth site because it may take some time for SCS clients to develop trust with peers and health staff to seek medical advice [44].

For all analyses, we ran a Durbin-Watson test [71, 72] and generated autocorrelation plots to check for correlation between data points over time. We used the Newey-West standard errors to account for autocorrelation identified using plots of residuals [73]. All analyses were conducted using SAS version 9.4 (Cary, NC) and R version 4.2.3.

The McGill University Research Ethics Board (A08-E53-19B; first approval date: 2019/08/26) and Quebec Access to Information Commission (1026586-S; 2021/09/20) approved the protocol. We rounded all frequencies to the nearest multiple of 5 or 10, as required by the *Institut de la Statistique du Québec*. Due to the retrospective nature of this study, informed consent was waived.

## Results

Table 1 shows the demographic characteristics of the study cohort. Nearly two-thirds (66.41%) of people who injected drugs in Montreal during the observation period were male, and the average age of individuals at the time of inclusion was 41.84 years. In the 1-year period preceding cohort inclusion, 10.92% of individuals were hospitalized and 7.34% visited the emergency department at least once. About 3.76% of the cohort were first identified as people who inject drugs from an OAT dispensation, and around 1.76% of the cohort had concurrent diagnosis of IRI at the time of cohort inclusion. A large majority (81.73%) of individuals were covered under Quebec’s public prescription drug insurance plan at some point over the course of follow-up.

**Table 1:** Characteristics of the study cohort^1^.

Demographic^2^	
Age (Mean & SD)	41.84 (13.38)
Male (%)	21,170 (66.41%)
Health Service Utilization in year before identification as person who inject drugs noted in administrative data^3^	
Hospitalization (Yes)	3,480 (10.92%)
Emergency Department Visit (Yes)	2,340 (7.34%)
Physician Visits	
0	24,490 (76.82%)
1–10	4,450 (13.96%)
11+	2,935 (9.21%)
OAT Initiation^4^	1200 (3.76%)
Coverage under the Public Prescription Drug Insurance Plan^5^	26,055 (81.73%)
IRI at cohort entry	560 (1.76%)

^1^ The numbers have been rounded to the nearest 5 or 10 in accordance with the *Centre d’accès aux données de recherche de l’Institut de la statistique du Québec* (CADRISQ) regulations on data confidentiality and presentation of research outputs.

^2^ Measured at baseline when the individual is identified as a person who injects drugs

^3^ Measured over the 1-year period before identification as a person who injects drugs

^4^ Initial identification as a person who injects drugs through records of OAT in prescription drug claims

^5^ Indicates coverage under the public prescription drug insurance plan at some point over the course of follow-up

Note: The results in this Table were compiled using data from the © Government of Québec (Research file publication date: 2009–2019; Data use approval date: 20 September 2021).

### Main analysis

**Fig 1:**
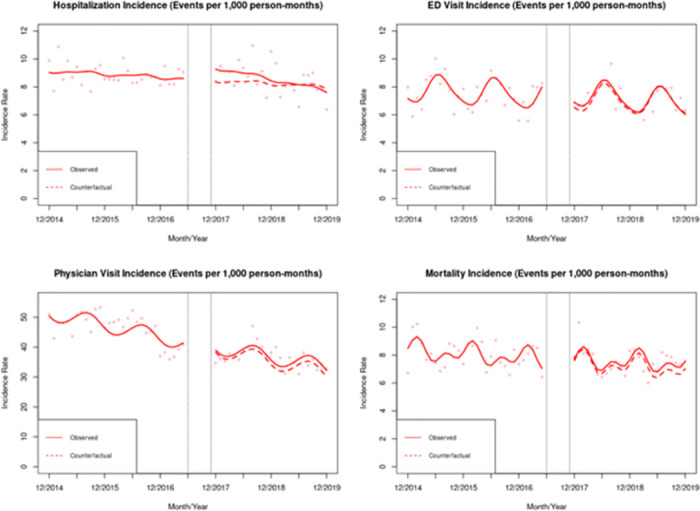
Observed IRI incidence rate and counterfactuals. Note: The results in this Figure were compiled using data from the © Government of Québec (Research file publication date: 2009–2019; Data use approval date: 20 September 2021).

Table 2 and Fig 1 summarize the results from the ITS analysis on the incidence of injection-related bacterial infections. After the implementation of the four SCS, we observed a level increase (${\hat{\beta }}_{2}$) in the monthly incidence rates of hospitalizations (0.97; 95% CI: 0.26, 1.68). Compared to pre-intervention trends (prior to June 2017), there was a negative post-intervention trend (${\hat{\beta }}_{3}$; after November 2017) in the monthly incidence rates of hospitalizations (-0.05; 95% CI: -0.08, -0.02), with limited and non-significant declining trend in ED visits (-0.02; 95% CI: -0.05, 0.02). No changes in level and trend were observed for IRI mortality. In our analysis using secondary outcomes, we observed a trend change (${\hat{\beta }}_{3}$) in the incidence of hospitalizations involving surgery (-0.03; 95% CI: -0.06, 0.00) but no effect on the average length of IRI hospitalizations (-0.09; 95% CI: -0.79, 0.61; S7 Table).

**Table 2:** Parameter estimates and 95% CI for the rates of injection-related infections from the interrupted time series model.

	Hospitalizations	Emergency Department Visits	Physician visits	IRI Mortality
Intercept (${\hat{\beta }}_{0}$)	**9.17 (8.87, 9.46)**	**7.91 (7.51, 8.30)**	**51.90 (48.60, 55.19)**	**8.41 (7.87, 8.95)**
Time (${\hat{\beta }}_{1}$)	**-0.02 (-0.04, 0.00)**	-0.02 (-0.04, 0.00)	**-0.34 (-0.54, -0.13)**	-0.02 (-0.05, 0.00)
Level change (${\hat{\beta }}_{2}$)	**0.97 (0.26, 1.68)**	0.39 (-0.20, 0.99)	0.72 (-3.85, 5.29)	0.11 (-0.83, 1.05)
Trend change (${\hat{\beta }}_{3}$)	**-0.05 (-0.08, -0.02)**	-0.02 (-0.05, 0.02)	0.06 (-0.23, 0.34)	0.02 (-0.04, 0.07)

Note: The results in this table were compiled using data from the © Government of Québec (Research file publication date: 2009–2019; Data use approval date: 20 September 2021). Bolded terms indicate p-value < 0.05. For all the regression models, the unit for Time was in months. The regression coefficient for Time represents the pre-intervention slope for the outcome incidence rate (number of events per 1,000 person-months) associated with 1-month increase in time. Level change refers to change in the outcome incidence rate following the intervention. Trend change refers to the slope in the outcome incidence rate over time following the intervention.

Table 3 displays the predicted changes in the IRI incidence rate compared to the incidence rate under a counterfactual scenario. After 12 months of the opening of all four SCS, the observed incidence rates (cases per 1,000 person-months) for IRI hospitalizations, ED visits, and physician visits were 0.37 cases higher, 0.17 cases higher, and 1.40 cases higher, respectively than what would have been expected if the SCS had not been implemented (relative effect changes: 4.45%, 2.57%, and 3.90%, respectively). After 24 months of the opening of the four sites, the observed incidence rates (cases per 1,000 person-months) for hospitalizations, ED visits, and physician visits were 0.23 cases lower, 0.06 cases lower, and 2.08 cases higher than what would have been expected, respectively, if the SCS had not been implemented (-2.96%, -0.86%, and 6.53% change, respectively).

**Table 3:** Absolute and relative effects of SCS on the incidence of IRI and their 95% confidence interval.

	Time since Implementation	Predicted Incidence Rate	Counterfactual Incidence Rate	Absolute Change	Relative Change (%)
Hospitalization	12 months	8.60	8.23	0.37	4.45%
	(11/2018)	(8.27, 8.93)	(7.90, 8.56)	(-0.30, 1.03)	(-3.45, 13.01)
	24 months	7.77	8.00	-0.23	-2.96%
	(11/2019)	(7.36, 8.18)	(7.60, 8.41)	(-1.05, 0.58)	(-12.52, 7.63)
Emergency Department Visits	12 months	6.76	6.59	0.17	2.57%
	(11/2018)	(6.46, 7.06)	(6.29, 6.89)	(-0.43, 0.76)	(-6.18, 12.15)
	24 months	6.32	6.38	-0.06	-0.86%
	(11/2019)	(5.82, 6.83)	(5.87, 6.88)	(-1.06, 0.95)	(-15.46, 16.25)
Physician visits	12 months	37.35	35.95	1.40	3.90%
	(11/2018)	(35.72, 38.97)	(34.32, 37.57)	(-1.85, 4.65)	(-4.92, 13.55)
	24 months	33.97	31.89	2.08	6.53%
	(11/2019)	(32.31, 35.62)	(30.23, 33.54)	(-1.23, 5.39)	(-3.66, 17.83)
IRI Mortality	12 months	7.24	6.92	0.32	4.50%
	(11/2018)	(6.82, 7.65)	(6.51, 7.34)	(-0.52, 1.14)	(-7.06, 17.53)
	24 months	7.14	6.63	0.51	7.69%
	(11/2019)	(6.55, 7.73)	(6.04, 7.22)	(-0.68, 1.70)	(-9.36, 28.08)

Note: The results in this Table were compiled using data from the © Government of Québec (Research file publication date: 2009–2019; Data use approval date: 20 September 2021).

### Secondary analysis

**Fig 2:**
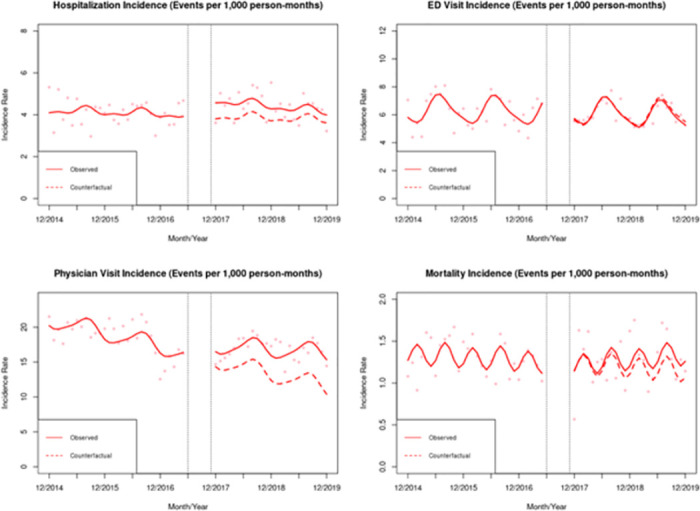
Observed SSTI incidence rate and counterfactuals.

Table 4 and Fig 2 summarize the results from the ITS analysis on the incidence of skin and soft tissue infections, specifically. After the implementation of the four SCS, we observed a level increase (${\hat{\beta }}_{2}$) in the monthly incidence rates of hospitalizations (0.78; 95% CI: 0.27, 1.28) and physician visits (2.05; 95% CI: 0.14, 3.96). Compared to pre-intervention trends (prior to June 2017), there was a small negative post-intervention trend (${\hat{\beta }}_{3}$; after November 2017) in the monthly incidence rate of hospitalizations (-0.02; 95% CI: -0.03, 0.00). Trend changes in ED visits (-0.01; 95% CI: -0.04, 0.01) remained limited, but there was a stronger positive trend in physician visits (0.12; 95% CI: 0.00, 0.23). No changes in level and trend were observed for the SSTI mortality. In our analysis with secondary outcomes, we observed a trend change (${\hat{\beta }}_{3}$) in the incidence of hospitalizations involving surgery (-0.01; 95% CI: -0.02, 0.00) but no effect on the average length of SSTI hospitalization stay (-0.05; 95% CI: -0.41, 0.32; S8 Table).

**Table 4:** Parameter estimates and 95% CI for the rates of skin and soft tissue infections from the interrupted time series model.

	Hospitalizations	Emergency Department Visits	Physician visits	SSTI Mortality
Intercept	**4.25**	**6.41**	**21.35**	**1.36**
(${\hat{\beta }}_{0}$)	**(4.00, 4.50)**	**(6.10, 6.73)**	**(20.03, 22.68)**	**(1.17, 1.55)**
Time	-0.01	-0.01	-0.16	0.00
(${\hat{\beta }}_{1}$)	(-0.02, 0.00)	(-0.02, 0.01)	(-0.24, -0.09)	(-0.01, 0.00)
Level change	**0.78**	0.12	**2.05**	-0.01
(${\hat{\beta }}_{2}$)	**(0.27, 1.28)**	(-0.33, 0.56)	**(0.14, 3.96)**	(-0.14, 0.12)
Trend change	-0.02	-0.01	**0.12**	0.01
(${\hat{\beta }}_{3}$)	(-0.03, 0.00)	(-0.04, 0.01)	**(0.00, 0.23)**	(0.00, 0.02)

Table 5 displays the predicted changes in the SSTI incidence rate compared to the incidence rate under a counterfactual scenario. After 12 months of the opening of all four SCS, the observed incidence rates (cases per 1,000 person-months) for SSTI hospitalizations, ED visits, and physician visits were 0.59 cases higher, 0.05 cases lower, and 3.44 cases higher, respectively than what would have been expected if the SCS had not been implemented (relative effect changes: 15.57%, -0.91%, and 25.87%, respectively). Similarly, after 24 months of the opening of the four sites, the observed incidence rates (cases per 1,000 person-months) for hospitalizations, ED visits, and physician visits were 0.39 cases higher, 0.22 cases lower, and 4.83 cases higher than what would have been expected, respectively, if the SCS had not been implemented (10.77%, -3.89%, and 42.63% change, respectively).

**Table 5:** Absolute and relative effects of SCS on the incidence of SSTI and their 95% confidence interval.

	Time since Implementation	Predicted Incidence Rate	Counterfactual Incidence Rate	Absolute Change	Relative Change (%)
Hospitalization	12 months	4.34	3.75	0.59	15.57%
	(11/2018)	(4.04, 4.64)	(3.45, 4.05)	(-0.02, 1.19)	(-0.43, 34.36)
	24 months	4.05	3.66	0.39	10.77%
	(11/2019)	(3.81, 4.29)	(3.42, 3.89)	(-0.08, 0.87)	(-2.11, 25.44)
Emergency Department Visits	12 months	5.76	5.81	-0.05	-0.91%
	(11/2018)	(5.47, 6.05)	(5.53, 6.10)	(-0.63, 0.52)	(-10.27, 9.41)
	24 months	5.53	5.75	-0.22	-3.89%
	(11/2019)	(5.24, 5.82)	(5.46, 6.04)	(-0.80, 0.36)	(-13.31, 6.53)
Physician visits	12 months	16.72	13.29	**3.44**	**25.87%**
	(11/2018)	(15.70, 17.75)	(12.26, 14.31)	**(1.39, 5.49)**	**(9.69, 44.74)**
	24 months	16.15	11.32	**4.83**	**42.63%**
	(11/2019)	(15.36, 16.95)	(10.53, 12.12)	**(3.23, 6.42)**	**(26.68, 60.99)**
SSTI Mortality	12 months	1.15	1.06	0.09	8.27%
	(11/2018)	(1.01, 1.28)	(0.93, 1.19)	(-0.17, 0.35)	(-14.61, 37.61)
	24 months	1.20	1.02	0.19	18.37%
	(11/2019)	(1.06, 1.34)	(0.88, 1.16)	(-0.09, 0.46)	(-7.81, 52.79)

### Sensitivity analyses

When we conducted sensitivity analyses assuming early effect (June 2017) of SCS (S3 and S5 Tables), we observed level changes (${\hat{\beta }}_{2}$) in hospitalization (1.40; 95% CI: 0.78, 2.01) and ED visit (0.98; 95% CI: 0.37, 1.59) rates for IRI. Similarly, we observed level changes (${\hat{\beta }}_{2}$) in hospitalization (0.95; 95% CI: 0.63, 1.27) and ED visit (0.53; 95% CI: 0.01, 1.05) rates for SSTI. Further, compared with the pre-intervention trends (prior to June 2017), there were declines in post-intervention trends (${\hat{\beta }}_{3}$) in hospitalization (-0.06; 95% CI: -0.09, -0.02) and ED visit (-0.04; 95% CI: -0.08, -0.01) rates for IRI. Relatedly, there were declines in post-intervention trends (${\hat{\beta }}_{3}$) in hospitalization (-0.02; 95% CI: -0.04, 0.00) and ED visit (-0.03; 95% CI: -0.06, 0.00) rates for SSTI. Meanwhile, we observed increases in post-intervention trends for physician visits to treat SSTI (0.14; 95% CI: 0.04, 0.23) but not for IRI. When we assumed late intervention effect (November 2017) of SCS (S4 and S6 Tables), there was a negative post-intervention trend (${\hat{\beta }}_{3}$) in hospitalizations (-0.08; 95% CI: -0.13, -0.04) and ED visits (-0.04; 95% CI: -0.08, -0.01) for IRI as well as in hospitalizations (-0.04; 95% CI: -0.05, -0.02) and ED visits (-0.03; 95% CI: -0.05, -0.01) for SSTI. There was a similar positive trend effect for physician visits for SSTI (0.08; 95% CI: 0.01, 0.16), but no level changes as observed using the November 2017 implementation date.

Additional findings from sensitivity analyses pertaining to secondary analysis with SSTI as the outcome are presented in Supplementary Materials.

## Discussion

To our knowledge, this was the first study to evaluate the population-level effects of supervised consumption sites on injection-related infection outcomes and health service use in people who inject drugs. We observed a moderately declining trend over time in the rates of hospitalizations for IRI following the implementation of the SCS. At the same time, we observed positive effects on the level and trends of physician visits for SSTI following the implementation of SCS. Montreal’s four SCS did not have an impact on the average length of hospitalization stay, but there was a small decline in the incidence of hospitalizations involving surgical procedures trends post-intervention. These findings suggest that SCS may have modest protective effects against more serious and costly IRI outcomes, with some evidence of their role in promoting early health-seeking behaviours.

A few factors may explain the declining trend observed in hospitalizations and, to a limited extent, in ED visits. With efforts to improve overall injection safety as well as prevent and manage infectious disease transmission among people who inject drugs taking place within SCS settings [74], the risk of more serious cases of IRI may have decreased over time. In addition, Montreal’s SCS operate under the “saturation strategy”, which encourages clients to gather as much equipment as needed to ensure each injection is performed with new materials [75]. With such strategy in place, the overall incidence of injection-related bacterial infections, including those not treated in healthcare system settings, may have decreased among people who inject drugs due to lower exposure to injection-related risk behaviours [76–78].

Our findings also complement an earlier study on Vancouver’s SCS, which concluded that SCS prevent lengthy and costly hospitalizations for IRI among clients who utilized the SCS services [37]. Although the magnitude of our study findings is smaller than that observed in Vancouver, this is partly because our study included people who inject drugs who did not utilize SCS. Further, SCS in British Columbia (BC) have hundreds of thousands of visits by over 5,000 unique individuals every year [79, 80], compared with Montreal’s SCS which report tens of thousands of visits by around 1,000 unique individuals annually [75]. The Vancouver sites in operation at the time of the study had longer operating hours (e.g., InSite: 9 AM-2 AM and Dr. Peter Centre: 24 hours) [81, 82], whereas none of the Montreal sites operate on a 24/7 basis [83]. Further, SCS in BC have nurses and medically trained staff on site who can refer clients to external healthcare services [84]. The largest SCS in Vancouver (i.e., InSite) refers thousands of clients to health and social care providers every month (5,125 referrals as of October 2023). Montreal’s four SCS combined made fewer than 100 referrals during the same period (Note: The number of referrals was suppressed for L’Anonyme, Cactus, and Spectre de Rue due to number of cases < 10.) [85].

Continuing our comparison with earlier studies in BC, the smaller effect of SCS on IRI and SSTI incidence observed in our study may be a consequence of differences in clinical capacity. Montreal’s four SCS have lacked consistent nursing staff since their opening in 2017 [86], limiting their healthcare interventions (e.g., wound care and preventive care) and medical referrals. For example, Dopamine has operated without nursing staff, and Cactus has operated with part-time nurses on site irregularly [86]. The potential impact of understaffing at these sites is reinforced by fewer than 10 referrals made to health and social services (e.g., wound care and primary healthcare) per month across all four sites [85]. The lack of medically trained professionals means that the sites may not be able to connect clients with the needed health care services [87]. To realize the full potential of Montreal’s SCS on injection-related bacterial infections, permanent placement of professional healthcare practitioners may be needed [88].

In the absence of medical professionals, relationships with peer staff at the SCS may explain the impacts observed in our study. People who inject drugs report they feel safe disclosing health concerns to peer workers early in diseases’ progression [89–91]; and peer support may encourage clients to seek medical care sooner [92]. Our findings also remain consistent with earlier studies conducted in other parts of the world [93, 94], which concluded lower likelihood of ED visits and hospitalizations but modestly higher likelihood of injection-related infections associated with SCS, owing to earlier diagnosis and treatment as well as increased support to clients and screening for these infections [95]. Thus, a more “personalized” and “de-medicalized” approach to SCS operations may encourage health service use for IRI and other injection-related harms even where medical oversight is missing [96, 97].

In addition, the full effect of the SCS on IRI incidence may not have been realized due to low SCS utilization by the people who inject drugs. According to Quebec Provincial Public Health Institute’s surveillance study, only 34.2% of people who inject drugs living in Montreal in 2018 injected in SCS settings in the previous six months [6]. While the four sites witnessed increases in the number of visits and clients in the two-year period following their openings [75]; Julien Montreuil, co-creator of the L’Anonyme site, notes stigma around drug use continues to discourage people who inject drugs from utilizing the city’s SCS [98]. Moreover, many people who inject drugs use SCS infrequently, which may limit the opportunities to develop the relationships needed for clients to confide in and seek health advice from site staff [87].

Our study has a few limitations. First, we used the Quebec provincial socio-sanitary region code to ascertain IRI cases among people who inject drugs in Montreal, which includes the regions within Montreal Island that are not served by SCS. A more localized approach comparing residents of neighborhoods with SCS to those without may have yielded stronger signals of their effectiveness. Our approach may have tempered the effect of SCS on IRI incidence, potentially leading to bias towards the null. However, with evidence of greater health seeking behaviours (i.e., greater rate of physician visits) and reduction of severe IRI cases, our results provide a positive albeit conservative estimate of the effect of the SCS. Second, by using administrative data, we were only able to include individuals who interacted with the healthcare system. While this may have underestimated target population and incidence of IRI among people who inject drugs in Montreal, our findings demonstrate evidence of health seeking behaviour and higher health service use among those who did use healthcare services. Third, the number of IRI cases claimed in physician visits may be underestimated as physicians are not required to provide ICD-9 codes in Quebec. To capture as many outpatient claims for IRI, we used the act codes that indicated procedures on skin and soft tissues in microbiology or infectious disease units. At the same time, there is no indication of changes in how physicians claimed these visits, that may otherwise explain differences in pre- vs. post-implementation level and trend changes observed. Fourth, the public prescription drug plan database captures only those who are publicly insured, which may have excluded some people who inject drugs in Montreal. However, recent surveillance on the Quebec population of people who inject drugs between 2011 and 2018 demonstrated that only 7.5% of people who inject drugs had their principal source of income through employment [6]. This employment statistic combined full-time, part-time, and contractual employment, which may indicate that even fewer individuals had access to employer sponsored drug insurance. Relatedly, over 70% of people who inject drugs indicated that their principal source of income came from social assistance or support and disability pension [6], which fall under the last-resort financial assistance. Therefore, the public prescription drug plan database captured the majority of people who inject drugs in Montreal, and it is unlikely that a large proportion of the people who inject drugs were systematically excluded using the administrative data algorithm.

## Conclusion

After the implementation of Montreal’s four SCS, there was a modest declining post-intervention trend for the incidence of hospitalizations, including those requiring surgical procedures. At the same time, the incidence rates of physician visits and hospitalizations for SSTI increased immediately after the implementation of Montreal’s four SCS. These results suggest that SCS may prevent progression of IRI to serious complications. The effects of Montreal’s SCS may be an underestimate owing to operational challenges and low uptake of SCS services by people who inject drugs. The full benefits of SCS on IRI may be materialized through additional on-site services and peer support that connect people who inject drugs with external health and social care services.

## Supporting information

S1 Text Acknowledgement of data owners in the official language of Québec. (DOCX)

S1 Table Diagnostic codes for conditions that identify people who inject drugs. Note: The * indicates that the code begins with the preceding alphanumeric characters. OAT categorized as “Others” includes diamorphine hydrochloride. Under “Other” drug misuse, the ICD-9 codes include drug induced mental health disorders; dependence on hallucinogens or combinations of drugs; and poisoning by drugs, medicinals, and biological substances. The ICD-10 codes corresponding to “Other” drug misuse include polysubstance drug use and poisoning by drugs, medicaments and biological substances (e.g., hallucinogens and psychotropic drugs). Abbreviations: ICD = International Classification of Diseases; OAT = Opioid Agonist Treatment; DIN = Drug Identification Number; PIN = Product Identification Number.
(DOCX)

S2 Table Diagnostic codes for injection-related infections. Note: The * indicates that the code begins with the preceding alphanumeric characters.
(DOCX)

S3 Table Parameter estimates and 95% confidence interval for injection-related infections from the interrupted time series model assuming early effect of supervised consumption sites in June 2017 (primary outcomes). Note: Bolded terms indicate p-value < 0.05. For all the regression models, the unit for Time was in months. The regression coefficient for Time represents the pre-intervention slope for the outcome incidence rate (number of events per 1,000 person-months) associated with 1-month increase in time. Level change refers to change in the outcome incidence rate following the intervention. Trend change refers to the slope in the outcome incidence rate over time following the intervention.
(DOCX)

S4 Table Parameter estimates and 95% confidence interval for injection-related infections from the interrupted time series model assuming late effect of supervised consumption sites in November 2017 (primary outcomes). Note: Bolded terms indicate p-value < 0.05. For all the regression models, the unit for Time was in months. The regression coefficient for Time represents the pre-intervention slope for the outcome incidence rate (number of events per 1,000 person-months) associated with 1-month increase in time. Level change refers to change in the outcome incidence rate following the intervention. Trend change refers to the slope in the outcome incidence rate over time following the intervention.
(DOCX)

S5 Table Parameter estimates and 95% confidence interval for skin and soft tissue infections from the interrupted time series model assuming early effect of supervised consumption sites in June 2017 (primary outcomes). Note: Bolded terms indicate p-value < 0.05. For all the regression models, the unit for Time was in months. The regression coefficient for Time represents the pre-intervention slope for the outcome incidence rate (number of events per 1,000 person-months) associated with 1-month increase in time. Level change refers to change in the outcome incidence rate following the intervention. Trend change refers to the slope in the outcome incidence rate over time following the intervention.
(DOCX)

S6 Table Parameter estimates and 95% confidence interval for skin and soft tissue infections from the interrupted time series model assuming late effect of supervised consumption sites in November 2017 (primary outcomes). Note: Bolded terms indicate p-value < 0.05. For all the regression models, the unit for Time was in months. The regression coefficient for Time represents the pre-intervention slope for the outcome incidence rate (number of events per 1,000 person-months) associated with 1-month increase in time. Level change refers to change in the outcome incidence rate following the intervention. Trend change refers to the slope in the outcome incidence rate over time following the intervention.
(DOCX)

S7 Table Parameter estimates and 95% confidence interval for injection-related infections from the interrupted time series model (secondary outcomes). Note: Bolded terms indicate p-value < 0.05. For all the regression models, the unit for Time was in months. The regression coefficient for Time represents the pre-intervention slope for the outcome associated with 1-month increase in time. The unit of measurement for the outcome was the number of days for the average length of IRI hospitalizations and incidence rate for IRI hospitalizations involving surgery (number of events per 1,000 person-months). Level change refers to change in the outcome following the intervention. Trend change refers to the slope in the outcome over time following the intervention.
(DOCX)

S8 Table Parameter estimates and 95% confidence interval for skin and soft tissue infections from the interrupted time series model (secondary outcomes). Note: Bolded terms indicate p-value < 0.05. For all the regression models, the unit for Time was in months. The regression coefficient for Time represents the pre-intervention slope for the outcome associated with 1-month increase in time. The unit of measurement for the outcome was the number of days for the average length of SSTI hospitalizations and incidence rate for SSTI hospitalizations involving surgery (number of events per 1,000 person-months). Level change refers to change in the outcome following the intervention. Trend change refers to the slope in the outcome over time following the intervention.
(DOCX)

S9 Table Absolute and relative effects of supervised consumption sites on hospitalizations associated with injection-related infections and their 95% confidence interval. (DOCX)

S10 Table Absolute and relative effects of supervised consumption sites on the hospitalizations associated with skin and soft tissue infections and their 95% confidence interval. (DOCX)

S1 Fig Observed incidence rate of injection-related infections and counterfactuals assuming early intervention effect in June 2017 (primary outcomes). (TIF)

S2 Fig Observed incidence rate of injection-related infections and counterfactuals assuming late intervention effect in November 2017 (primary outcomes). (TIF)

S3 Fig Observed incidence rate of skin and soft tissue infections and counterfactuals assuming early intervention effect in June 2017 (primary outcomes). (TIF)

S4 Fig Observed incidence rate of skin and soft tissue infections and counterfactuals assuming late intervention effect in November 2017 (primary outcomes). (TIF)

S5 Fig Average duration of injection-related infection hospitalizations and incidence rate of injection-related infection hospitalizations involving surgery. (TIF)

S6 Fig Average duration of skin and soft tissue infection hospitalizations and incidence rate of skin and soft tissue infection hospitalizations involving surgery. (TIF)

S7 Fig Average duration of injection-related infection hospitalizations and incidence rate of injection-related infection hospitalizations involving surgery (early effect in June 2017). (TIF)

S8 Fig Average duration of injection-related infection hospitalizations and incidence rate of injection-related infection hospitalizations involving surgery (late effect in November 2017). (TIF)

S9 Fig Average duration of skin and soft tissue infection hospitalizations and incidence rate of skin and soft tissue infection hospitalizations involving surgery (early effect in June 2017). (TIF)

S10 Fig Average duration of skin and soft tissue infection hospitalizations and incidence rate of skin and soft tissue infection hospitalizations involving surgery (late effect in November 2017). (TIF)
