# Supplementary material for: The effect of Montreal’s supervised consumption sites on injection-related infections among people who inject drugs: An interrupted time series
Source: PLoS One. 2024 Aug 27;19(8):e0308482. doi: 10.1371/journal.pone.0308482 (PMC11349102; doi:10.1371/journal.pone.0308482)
Supplement: S1 Text — (DOCX) [file pone.0308482.s001.docx]

**S1 Text. Acknowledgement of data owners in the official language of Québec**

Une partie de la compilation est effectuée à partir de données provenant du © Gouvernement du Québec (année de la publication du Fichier de recherche: 2009-2019). Le Gouvernement du Québec n’est pas responsable des compilations ni de l’interprétation des résultats produits à l’aide du Fichier de recherche.
