# Supplementary material for: The effect of Montreal’s supervised consumption sites on injection-related infections among people who inject drugs: An interrupted time series
Source: PLoS One. 2024 Aug 27;19(8):e0308482. doi: 10.1371/journal.pone.0308482 (PMC11349102; doi:10.1371/journal.pone.0308482)
Supplement: S1 Table — Note: The * indicates that the code begins with the preceding alphanumeric characters. OAT categorized as “Others” includes diamorphine hydrochloride. Under “Other” drug misuse, the ICD-9 codes include drug induced mental health disorders; dependence on hallucinogens or combinations of drugs; and poisoning by drugs, medicinals, and biological substances. The ICD-10 codes corresponding to “Other” drug misuse include polysubstance drug use and poisoning by drugs, medicaments and biological substances (e.g., hallucinogens and psychotropic drugs). Abbreviations: ICD = International Classification of Diseases; OAT = Opioid Agonist Treatment; DIN = Drug Identification Number; PIN = Product Identification Number. (DOCX) [file pone.0308482.s002.docx]

**S1 Table. Diagnostic codes for conditions suggestive of injection drug use that identify people who inject drugs**

| **Drug misuse diagnoses** | **ICD-9** | **ICD-10** | **DIN/PINs** |
| --- | --- | --- | --- |
| Opiates | 3040*, 3047*, 3055*, 9650* | E850*, F11*, T400, T401, T402, T403, T404, T406, R781 |  |
| Cocaine | 3042*, 3056*, 970* | F14*, R782, T405 |  |
| Amphetamines | 3044*, 3057*, 9697* | F15*, T436 |  |
| Sedatives | 3041*, 9694*, 3054* | F13*, T423-T428 |  |
| Other | 292*, 3045*, 3046*, 3048*, 3049*, 3053*, 3059*, 6483*, 7960*, 9621*, 9658*, 9663*, 9664*, 9670*, 9684*, 9685*, 9696*, 9698*, 9699*, 970* | F19*, R784, T42*, T387, T408, T409, T412, T436-T439, T507, V6542, X62, Y12, Z715*, Z503* |  |
| Drug use |  | Z72.2  Z86.41 |  |
| Homelessness |  | Z59 |  |
| Fee item (OAT) | 39 |  |  |
| OAT | 45373 |  | Methadone: 00999792, 00999793, 66999990-67000020  Buprenorphine / Naloxone: 02295695, 02295709, 02408090, 02408104, 02424851, 02424878, 02453908, 02453916, 02468085,  02468093  Buprenorphine: 02242962, 02242963, 02242964, 66999994-66999996  Slow-release oral morphine: 22123346 – 22123349  Butrans: 02341174, 02341212, 02341220  iOAT: 66123367, 02146126, 22123340  Others: 00655619, 00655627, 00781460, 00781479, 00999776 |

Note: The * indicates that the code begins with the preceding alphanumeric characters. OAT categorized as “Others” includes diamorphine hydrochloride. Under “Other” drug misuse, the ICD-9 codes include drug induced mental health disorders; dependence on hallucinogens or combinations of drugs; and poisoning by drugs, medicinals, and biological substances. The ICD-10 codes corresponding to “Other” drug misuse include polysubstance drug use and poisoning by drugs, medicaments and biological substances (e.g., hallucinogens and psychotropic drugs).

Abbreviations: ICD = International Classification of Diseases; OAT = Opioid Agonist Treatment; DIN = Drug Identification Number; PIN = Product Identification Number
