# Supplementary material for: The effect of Montreal’s supervised consumption sites on injection-related infections among people who inject drugs: An interrupted time series
Source: PLoS One. 2024 Aug 27;19(8):e0308482. doi: 10.1371/journal.pone.0308482 (PMC11349102; doi:10.1371/journal.pone.0308482)
Supplement: S2 Table — Note: The * indicates that the code begins with the preceding alphanumeric characters. (DOCX) [file pone.0308482.s003.docx]

**S2 Table. Diagnostic codes for injection-related infections**  [1]

| Version | IRI List |
| --- | --- |
| ICD-9 | Skin and soft tissue infections  Erysipelas (035)  Gas gangrene (0400*)  Intracranial abscess (324*)  Phlebitis and thrombophlebitis (451*)  Other suppurative peritonitis (5672)  Peritoneal abscess (56722)  Retroperitoneal infections (56731, 56738)  Abscess of intestine (5695*)  Abscess of liver (5720*)  Acute pyelonephritis (5901*)  Cellulitis (681*)  Other cellulitis and abscess (682*)  Other specified diseases of hair and hair follicles (7048)  Ulcer of lower limbs (7071*)  Chronic ulcer of other specified sites (7078*)  Chronic ulcer of unspecified (7079*)  Other specified disorders of skin (7098*)  Panniculitis specified as affecting neck (7236*)  Necrotizing fasciitis (72886)  Panniculitis (7293)  Panniculitis, unspecified site (72930)  Panniculitis, other site (72939)  Gangrene (7854*)  Endocarditis  Candidiasis of other specified sites (1128)  Candidal endocarditis (11281)  Acute and subacute endocarditis (421*)  Other diseases of endocardium (424*)  Bacteremia or sepsis  Septicemia (038*)  Pulmonary embolism and infarction (4151)  Septic pulmonary embolism (41512)  Other and unspecified myocarditis (4229)  Septic myocarditis (42292)  Septic arterial embolism (449*)  Shock without mention of trauma, including septic shock (7855*)  Bacteremia (7907*)  Systemic inflammatory response syndrome (9959)  Systemic inflammatory response syndrome, unspecified (99590)  Sepsis (99591)  Severe sepsis (99592)  Osteomyelitis  Acute osteomyelitis (7300*)  Chronic osteomyelitis (7301*)  Unspecified osteomyelitis (7302*)  Unspecified infection of bone (7309*)  Myositis  Myalgia and myositis, unspecified (7291*) |
| ICD-10 | Skin and soft tissue infections  Phlebitis and thrombophlebitis (I80*)  Non-pressure chronic ulcer of lower limb, not elsewhere classified (L97*)  Other specified disorders of the skin and subcutaneous tissue (L988)  Panniculitis, unspecified (M793*)  Gas gangrene (A480)  Intracranial abscess and granuloma (G06*)  Sequelae of inflammatory diseases of central nervous system (G09)  Abscess of intestine (K630)  Generalized (acute) peritonitis (K650)  Abscess of liver (K750)  Cutaneous abscess or furuncle (L02*)  Cellulitis (L03*)  Panniculitis affecting regions of neck and back, cervical region (M5402)  Necrotizing fasciitis (M726*)  Acute tubule-interstitial nephritis (N10)  Endocarditis  Candidal endocarditis (B376)  Acute and subacute endocarditis (I33*)  Non-rheumatic mitral valve disorders (I34*)  Non-rheumatic aortic valve disorders (I35*)  Non-rheumatic tricuspid valve disorders (I36*)  Non-rheumatic pulmonary valve disorders (I37*)  Endocarditis, valve unspecified (I38)  Endocarditis and heart valve disorders in diseases classified elsewhere (I39*)  Bacteremia or sepsis  Acute myocarditis (A40*)  Myocarditis in diseases classified elsewhere (A41*)  Pulmonary embolism without acute cor pulmonale (I269)  Infective myocarditis (I400)  Septic shock (R572)  Systemic inflammatory response syndrome of non-infectious origin (R651)  Systemic inflammatory response syndrome, unspecified (R659)  Osteomyelitis  Osteomyelitis (M86*)  Disorder of bone, unspecified (M899*)  Myositis  Myositis (M60*) |

Note: The * indicates that the code begins with the preceding alphanumeric characters.

REFERENCES

1. Janjua, N.Z., et al., *Identifying injection drug use and estimating population size of people who inject drugs using healthcare administrative datasets.* Int J Drug Policy, 2018. **55**: p. 31-39.
