# Supplementary material for: The effect of Montreal’s supervised consumption sites on injection-related infections among people who inject drugs: An interrupted time series
Source: PLoS One. 2024 Aug 27;19(8):e0308482. doi: 10.1371/journal.pone.0308482 (PMC11349102; doi:10.1371/journal.pone.0308482)
Supplement: S6 Table — Note: Bolded terms indicate p-value < 0.05. For all the regression models, the unit for Time was in months. The regression coefficient for Time represents the pre-intervention slope for the outcome incidence rate (number of events per 1,000 person-months) associated with 1-month increase in time. Level change refers to change in the outcome incidence rate following the intervention. Trend change refers to the slope in the outcome incidence rate over time following the intervention. (DOCX) [file pone.0308482.s007.docx]

**S6 Table. Parameter estimates and 95% confidence interval for skin and soft tissue infections from the interrupted time series model assuming late effect of supervised consumption sites in November 2017 (primary outcomes)**

|  | Hospitalizations | Emergency Department Visits | Physician Visits | SSTVI Mortality |
| --- | --- | --- | --- | --- |
| Intercept  ($\hat{\beta}_{0}$) | **4.06**  **(3.72, 4.39)** | **6.26**  **(5.95, 6.57)** | **21.02**  **(20.01, 22.04)** | **1.35**  **(1.18, 1.53)** |
| Time  ($\hat{\beta}_{1}$) | 0.01  (-0.01, 0.03) | 0.01  (-0.01, 0.03) | **-0.13**  **(-0.19, -0.07)** | 0.00  (-0.01, 0.00) |
| Level change  ($\hat{\beta}_{2}$) | 0.35  (-0.33, 1.02) | -0.21  (-0.67, 0.25) | 1.30  (-0.98, 3.58) | -0.01  (-0.17, 0.15) |
| Trend change  ($\hat{\beta}_{3}$) | **-0.04**  **(-0.05, -0.02)** | **-0.03**  **(-0.05, -0.01)** | **0.08**  **(0.01, 0.16)** | 0.01  (0.00, 0.02) |

Note: Bolded terms indicate p-value < 0.05. For all the regression models, the unit for Time was in months. The regression coefficient for Time represents the pre-intervention slope for the outcome incidence rate (number of events per 1,000 person-months) associated with 1-month increase in time. Level change refers to change in the outcome incidence rate following the intervention. Trend change refers to the slope in the outcome incidence rate over time following the intervention.
