# Supplementary material for: The effect of Montreal’s supervised consumption sites on injection-related infections among people who inject drugs: An interrupted time series
Source: PLoS One. 2024 Aug 27;19(8):e0308482. doi: 10.1371/journal.pone.0308482 (PMC11349102; doi:10.1371/journal.pone.0308482)
Supplement: S8 Table — Note: Bolded terms indicate p-value < 0.05. For all the regression models, the unit for Time was in months. The regression coefficient for Time represents the pre-intervention slope for the outcome associated with 1-month increase in time. The unit of measurement for the outcome was the number of days for the average length of SSTI hospitalizations and incidence rate for SSTI hospitalizations involving surgery (number of events per 1,000 person-months). Level change refers to change in the outcome following the intervention. Trend change refers to the slope in the outcome over time following the intervention. (DOCX) [file pone.0308482.s009.docx]

**S8 Table. Parameter estimates and 95% confidence interval for skin and soft tissue infections from the interrupted time series model (secondary outcomes)**

|  | Main Model | | Early Effect (06/2017) | | Late Effect (11/2017) | |
| --- | --- | --- | --- | --- | --- | --- |
|  | Average Length of SSTI Hospitalizations | SSTI Hospitalizations involving Surgery | Average Length of SSTI Hospitalizations | SSTI Hospitalizations involving Surgery | Average Length of SSTI Hospitalizations | SSTI Hospitalizations involving Surgery |
| Intercept  ($\hat{\beta}_{0}$) | **23.80**  **(20.65, 26.94)** | **1.57**  **(1.40, 1.74)** | **20.54**  **(19.32, 21.75)** | **3.91**  **(3.66, 4.16)** | **20.20**  **(18.56, 21.84)** | **3.84**  **(3.65, 4.03)** |
| Time  ($\hat{\beta}_{1}$) | **-0.19**  **(-0.33, -0.04)** | -0.01  (-0.01, 0.00) | **-0.12**  **(-0.18, -0.07)** | 0.00  (-0.01, 0.01) | **-0.09**  **(-0.17, -0.02)** | 0.00  (-0.01, 0.02) |
| Level change  ($\hat{\beta}_{2}$) | 2.22  (-2.21, 6.65) | 0.13  (-0.09, 0.35) | 2.65  (-0.53, 5.83) | 0.07  (-0.28, 0.42) | 1.72  (-3.82, 7.25) | -0.40  (-0.84, 0.03) |
| Trend change  ($\hat{\beta}_{3}$) | -0.05  (-0.41, 0.32) | **-0.01**  **(-0.02, 0.00)** | -0.08  (-0.51, 0.35) | **-0.04**  **(-0.06, -0.02)** | -0.12  (-0.69, 0.44) | **-0.04**  **(-0.06, -0.01)** |

Note: Bolded terms indicate p-value < 0.05. For all the regression models, the unit for Time was in months. The regression coefficient for Time represents the pre-intervention slope for the outcome associated with 1-month increase in time. The unit of measurement for the outcome was the number of days for the average length of SSTI hospitalizations and incidence rate for SSTI hospitalizations involving surgery (number of events per 1,000 person-months). Level change refers to change in the outcome following the intervention. Trend change refers to the slope in the outcome over time following the intervention.
