# Supplementary material for: The effect of Montreal’s supervised consumption sites on injection-related infections among people who inject drugs: An interrupted time series
Source: PLoS One. 2024 Aug 27;19(8):e0308482. doi: 10.1371/journal.pone.0308482 (PMC11349102; doi:10.1371/journal.pone.0308482)
Supplement: S9 Table — (DOCX) [file pone.0308482.s010.docx]

**S9 Table. Absolute and relative effects of supervised consumption sites on hospitalizations associated with injection-related infections and their 95% confidence interval**

|  | Time since Implementation | Predicted Value | Counterfactual Value | Absolute Change | Relative Change (%) |
| --- | --- | --- | --- | --- | --- |
| Average Length of Hospital Stay | 12 months  (11/2018) | 15.20  (10.25, 20.15) | 13.93  (8.98, 18.88) | 1.27  (-8.63, 11.16) | 9.11%  (-45.69, 124.3) |
|  | 24 months  (11/2019) | 12.70  (-0.58, 25.99) | 12.49  (-0.80, 25.77) | 0.21  (-26.36, 26.79) | -6.47%  (-102.3, 3352) |
| Hospitalizations Involving Surgery | 12 months  (11/2018) | 3.30  (3.01, 3.59) | 3.98  (3.69, 4.27) | -0.68  (-1.26, -0.09) | -17.06%  (-19.58, -2.55) |
|  | 24 months  (11/2019) | 2.95  (2.43, 3.47) | 3.99  (3.47, 4.50) | -1.04  (-2.07, 0.00) | -25.94%  (-45.95, 0.06) |
