# Supplementary material for: The effect of Montreal’s supervised consumption sites on injection-related infections among people who inject drugs: An interrupted time series
Source: PLoS One. 2024 Aug 27;19(8):e0308482. doi: 10.1371/journal.pone.0308482 (PMC11349102; doi:10.1371/journal.pone.0308482)
Supplement: S10 Table — (DOCX) [file pone.0308482.s011.docx]

**S10 Table. Absolute and relative effects of supervised consumption sites on the hospitalizations associated with skin and soft tissue infections and their 95% confidence interval**

|  | Time since Implementation | Predicted Value | Counterfactual Value | Absolute Change | Relative Change (%) |
| --- | --- | --- | --- | --- | --- |
| Average Length of Hospital Stay | 12 months  (11/2018) | 15.18  (11.27, 19.09) | 13.52  (9.61, 17.43) | 1.66  (-6.16, 9.49) | 12.30%  (-35.34, 98.72) |
|  | 24 months  (11/2019) | 12.39  (5.96, 18.82) | 11.28  (4.85, 17.71) | 1.11  (-11.75, 13.97) | 9.82%  (-66.34, 287.8) |
| Hospitalizations Involving Surgery | 12 months  (11/2018) | 1.29  (1.12, 1.45) | 1.30  (1.14, 1.47) | -0.02  (-0.35, 0.31) | -1.45%  (-23.61, 27.06) |
|  | 24 months  (11/2019) | 1.07  (0.80, 1.34) | 1.24  (0.97, 1.51) | -0.17  (-0.71, 0.38) | -13.44%  (-46.91, 38.74) |
