# Supplementary figures and images for: The effect of Montreal’s supervised consumption sites on injection-related infections among people who inject drugs: An interrupted time series
Source: PLoS One. 2024 Aug 27;19(8):e0308482. doi: 10.1371/journal.pone.0308482 (PMC11349102; doi:10.1371/journal.pone.0308482)

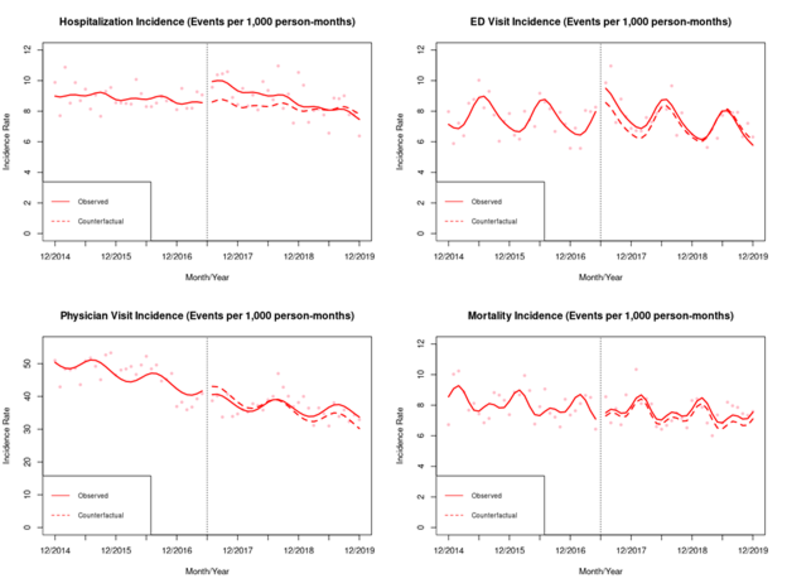

Supplement: S1 Fig — (TIF) [file pone.0308482.s012.tif]

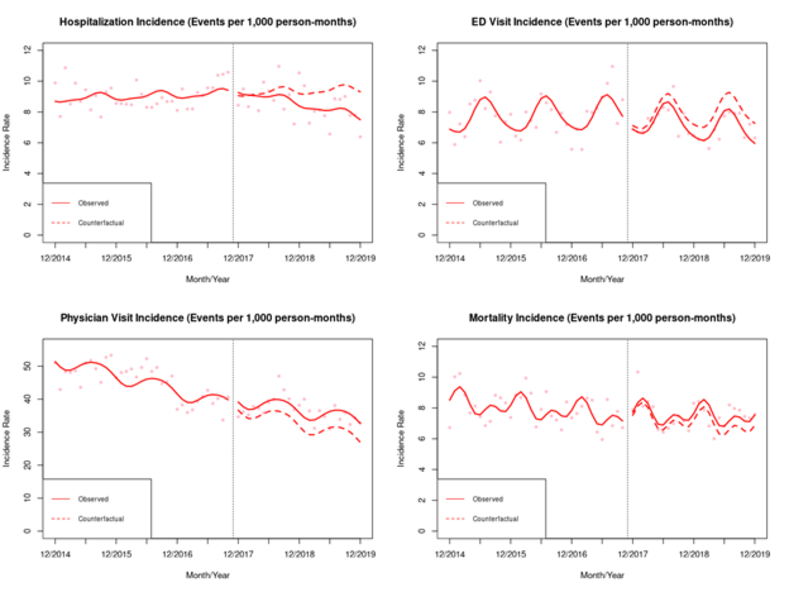

Supplement: S2 Fig — (TIF) [file pone.0308482.s013.tif]

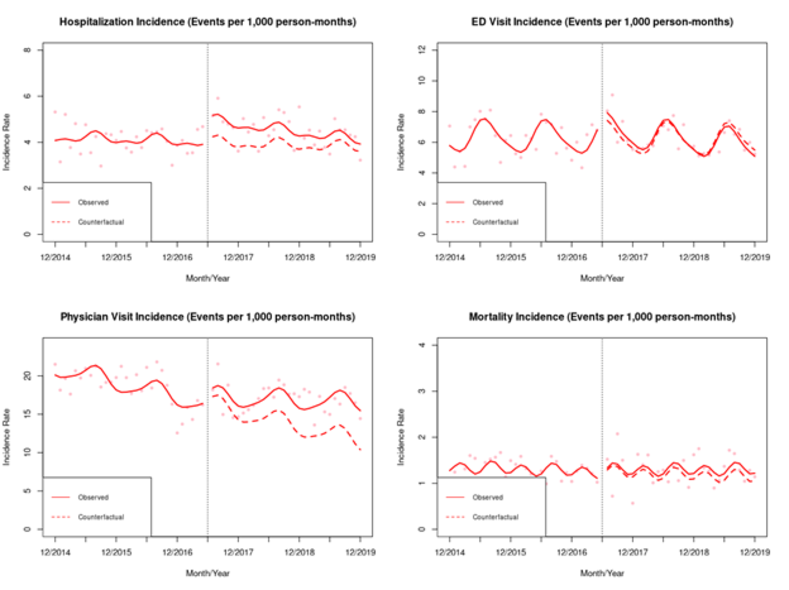

Supplement: S3 Fig — (TIF) [file pone.0308482.s014.tif]

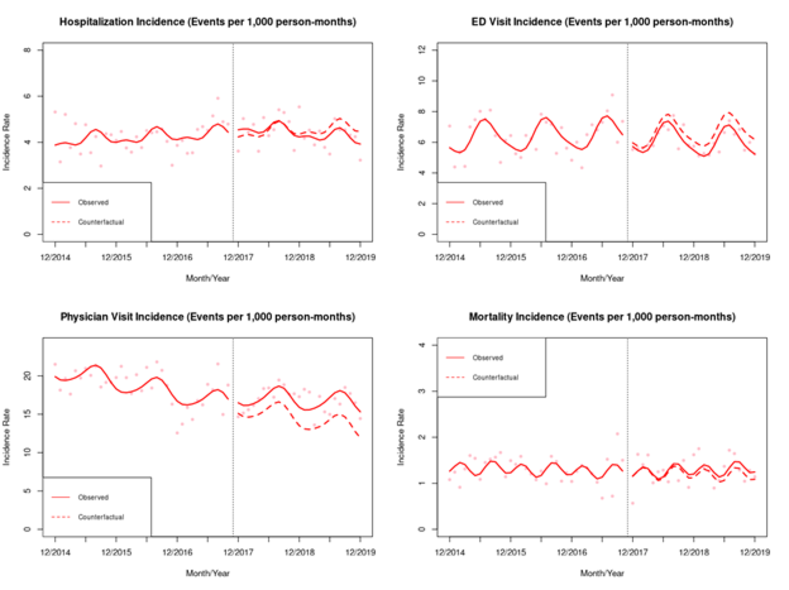

Supplement: S4 Fig — (TIF) [file pone.0308482.s015.tif]

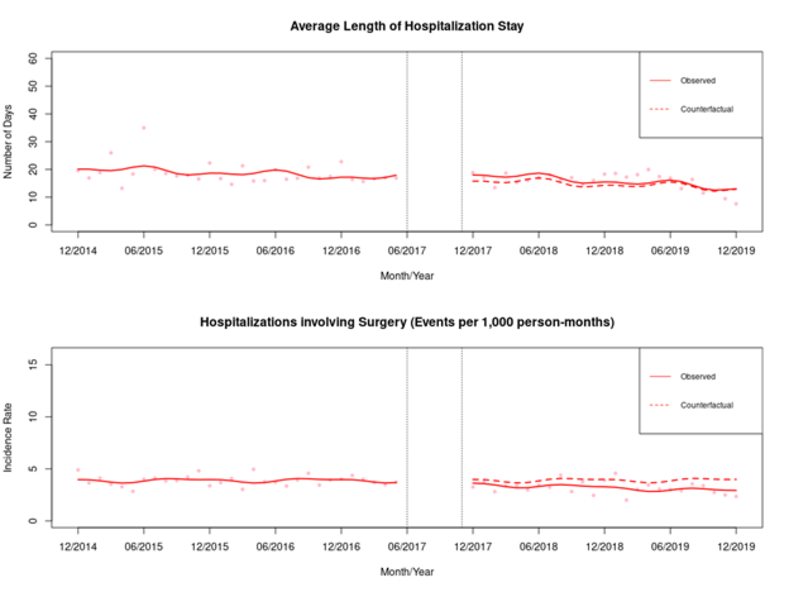

Supplement: S5 Fig — (TIF) [file pone.0308482.s016.tif]

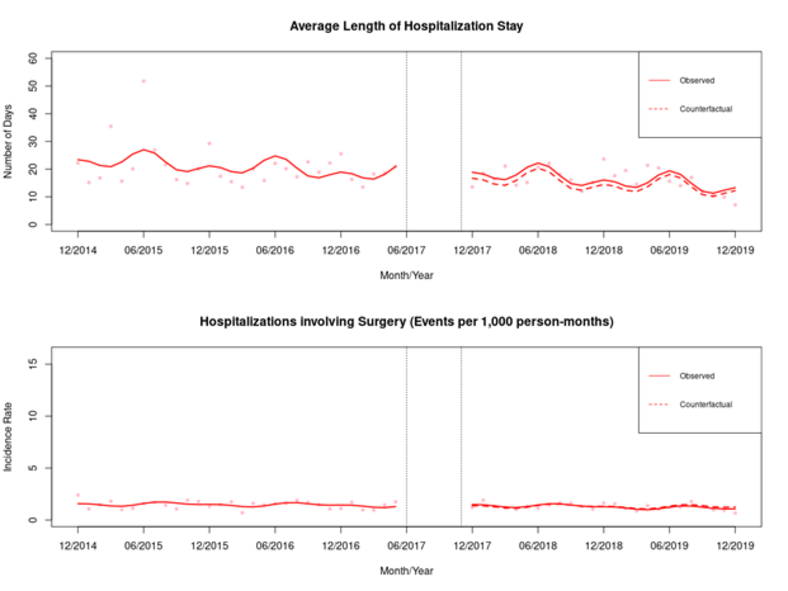

Supplement: S6 Fig — (TIF) [file pone.0308482.s017.tif]

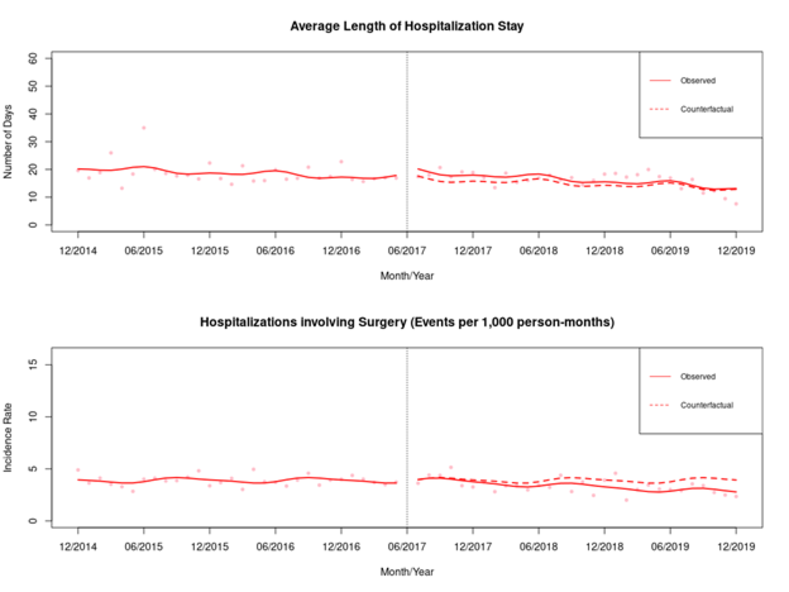

Supplement: S7 Fig — (TIF) [file pone.0308482.s018.tif]

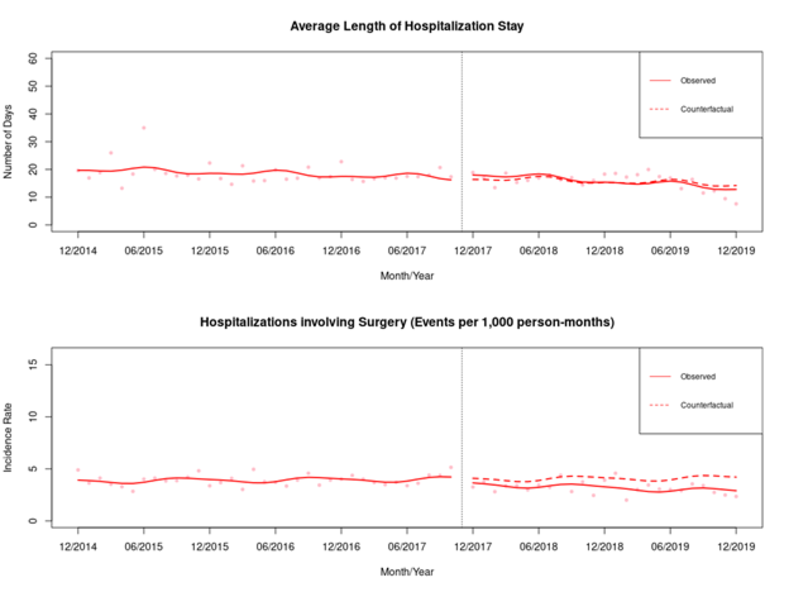

Supplement: S8 Fig — (TIF) [file pone.0308482.s019.tif]
